# Regulation of Ergosterol Biosynthesis in *Saccharomyces cerevisiae*

**DOI:** 10.3390/genes11070795

**Published:** 2020-07-15

**Authors:** Tania Jordá, Sergi Puig

**Affiliations:** Departamento de Biotecnología, Instituto de Agroquímica y Tecnología de Alimentos (IATA), Consejo Superior de Investigaciones Científicas (CSIC), Agustín Escardino 7, E-46980 Paterna, Valencia, Spain; tajorsan@alumni.uv.es

**Keywords:** ergosterol, sterol biosynthesis, sterol regulation, yeast, *Saccharomyces cerevisiae*, oxygen, iron

## Abstract

Ergosterol is an essential component of fungal cell membranes that determines the fluidity, permeability and activity of membrane-associated proteins. Ergosterol biosynthesis is a complex and highly energy-consuming pathway that involves the participation of many enzymes. Deficiencies in sterol biosynthesis cause pleiotropic defects that limit cellular proliferation and adaptation to stress. Thereby, fungal ergosterol levels are tightly controlled by the bioavailability of particular metabolites (e.g., sterols, oxygen and iron) and environmental conditions. The regulation of ergosterol synthesis is achieved by overlapping mechanisms that include transcriptional expression, feedback inhibition of enzymes and changes in their subcellular localization. In the budding yeast *Saccharomyces cerevisiae*, the sterol regulatory element (SRE)-binding proteins Upc2 and Ecm22, the heme-binding protein Hap1 and the repressor factors Rox1 and Mot3 coordinate ergosterol biosynthesis (*ERG*) gene expression. Here, we summarize the sterol biosynthesis, transport and detoxification systems of *S. cerevisiae*, as well as its adaptive response to sterol depletion, low oxygen, hyperosmotic stress and iron deficiency. Because of the large number of *ERG* genes and the crosstalk between different environmental signals and pathways, many aspects of ergosterol regulation are still unknown. The study of sterol metabolism and its regulation is highly relevant due to its wide applications in antifungal treatments, as well as in food and pharmaceutical industries.

## 1. Introduction

Sterols are essential components of eukaryotic cellular membranes that maintain membrane structural integrity, fluidity and permeability. They have further functions in regulating membrane-bound enzyme activity, lipid raft formation and function, substance transportation and cell cycle. The most important sterol in animals is cholesterol, which is additionally necessary as a precursor for the synthesis of vitamin D, bile acids and steroid hormones. Disturbed cholesterol homeostasis can lead to several human diseases, such as diabetes, atherosclerosis and neurodegeneration [1]. Plant sterols are called phytosterols, and the most notable are stigmasterol, sitosterol and campesterol. They are essential for plant growth and development, and play an important role in stress adaptation [2]. The main fungal sterol is ergosterol, which is regarded as a “fungal hormone” that can stimulate growth and proliferation. Ergosterol has recently been identified as an immunoactive lipid that induces host cells pyroptosis, a necrotic and inflammatory programmed cell death [3]. Significantly, recent studies have shown that ergosterol is essential for mitochondrial DNA maintenance in fungi, as cholesterol does in humans [4,5]. In fact, the pharmacological or genetic inhibition of ergosterol biosynthesis leads to the loss of mitochondrial DNA in *S. cerevisiae* unless exogenous ergosterol is added, highlighting the crosstalk between mitochondria and ergosterol biosynthesis [4]. Thus, ergosterol abundance is critical for yeast stress adaptation. For example, increased ergosterol levels have been associated with higher resistance to low temperature, freezing, low sugar, alcohol and oxidative stress. Conversely, hyperosmotic stress adaptation (such as salt or sorbitol treatment) leads to the decrease in ergosterol abundance [6,7]. Ergosterol levels are also important for the hypoxic and iron deficiency responses in *S. cerevisiae*, conditions in which the rate of ergosterol biosynthesis is limited by defects in specific enzymes that depend on oxygen and/or iron as an essential substrate or cofactor [8,9].

As a result of their versatile properties, sterols show wide applications in food and pharmaceutical industries [8]. The industrial production of ergosterol is achieved by yeast fermentation or extraction from fungal mycelia. Ergosterol, which is structurally analogous to cholesterol, has been used as a precursor of vitamin D2 and steroid hormone drugs. Intermediates of the ergosterol biosynthesis pathway, such as farnesyl diphosphate and squalene, are also economically interesting due to their use in terpenoid production, and for applications such as perfume ingredients, pharmaceuticals and advanced biofuels [10]. Moreover, ergosterol derivatives have significant antitumor and anti-HIV activities [11,12]. Remarkably, the ergosterol biosynthetic pathway represents the main target for the development of many antifungal agents because it is essential for fungal growth and viability, and its synthesis differs in particular steps from the major sterol-producing pathway in humans.

## 2. Ergosterol Synthesis, Uptake and Detoxification in *S. cerevisiae*

### 2.1. Ergosterol Biosynthesis in S. cerevisiae

**Figure 1:**
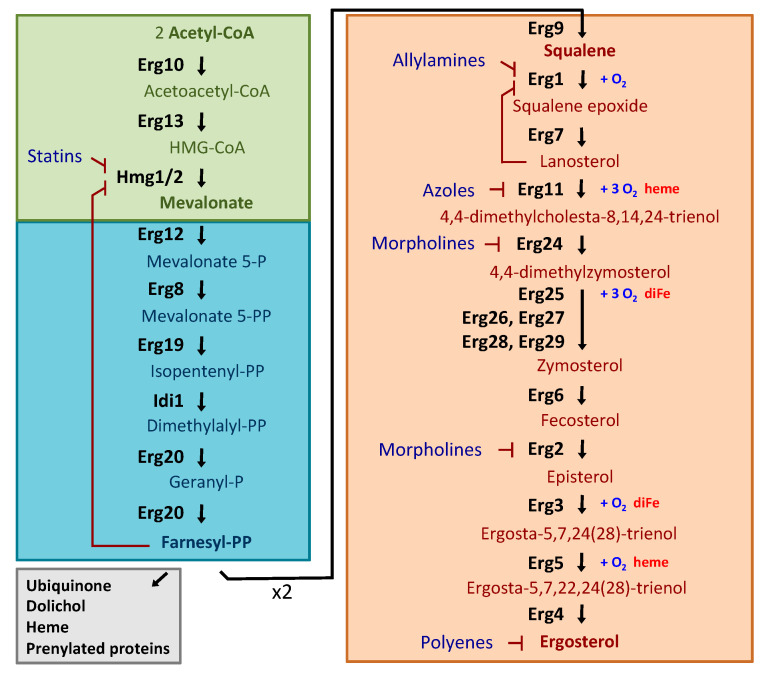
Ergosterol biosynthetic pathway in *S. cerevisiae*. The different color boxes represent the three modules into which the pathway can be divided: the green box is the mevalonate pathway, which occurs in the vacuole and mitochondria; the blue box consists of farnesyl pyrophosphate (farnesyl-PP) biosynthesis and is carried out in the vacuole; and the orange box contains the late pathway, which ends with ergosterol biosynthesis, and mainly takes place in the ER. In addition, farnesyl-PP is used in the synthesis of ubiquinone, dolichol, heme and prenylated proteins (gray box). Enzymes, intermediates, inhibitors and requirements of oxygen, heme and iron are indicated. Enzymes: Erg10, acetyl-CoA C-acetyltransferase; Erg13, HMG-CoA synthase; Hmg1/2, HMG-CoA reductase; Erg12, mevalonate kinase; Erg8, phosphomevalonate kinase; Mvd1/Erg19, mevalonate pyrophosphate decarboxylase; Idi1, Isopentenyl diphosphate Isomerase; Erg20, farnesyl pyrophosphate synthetase; Erg9, squalene synthase; Erg1, squalene epoxidase; Erg7, lanosterol synthase; Erg11 (Cyp51), lanosterol C-14 demethylase; Erg24, sterol C-14 reductase; Erg25, sterol C-4 methyloxydase; Erg26, sterol C-3 dehydrogenase (C4-decarboxylase); Erg27, sterol C-3 ketoreductase; Erg6, sterol C-24 methyltransferase; Erg2, sterol C-8 isomerase; Erg3, sterol C-5 desaturase; Erg5, sterol C-22 desaturase; Erg4, sterol C-24 reductase. Inhibitors: statins target Hmg1/2; allylamines inhibit Erg1; azoles inhibit Erg11; morpholines target Erg2 and, mainly, Erg24; polyenes bind ergosterol.

The budding yeast *S. cerevisiae* is a popular model organism to study intracellular sterol homoeostasis because the basic steps of yeast sterol metabolism and trafficking are conserved in other eukaryotic organisms. Under aerobic conditions, yeast cells do not incorporate exogenous sterols. Instead, they satisfy their sterol requirements by synthesizing their own ergosterol. Yeast ergosterol is synthesized through a highly conserved and complex pathway that can be divided into three modules (Figure 1) (reviewed in [6]). The first module is conserved across all eukaryotes and results in the formation of mevalonate from acetyl-coenzyme A (acetyl-CoA). The rate-limiting step of this stage is the reduction to mevalonate by the 3-hydroxy-3-methylglutaryl-coenzyme A (HMG-CoA) reductases (HMGR) Hmg1 and Hmg2 (Hmg1/2), which are derived from a single ancestral *HMGR* gene by gene duplication. The second module is carried out in the vacuole and involves the formation of farnesyl pyrophosphate (farnesyl-PP), which is also an important intermediate in the biosynthesis of ubiquinone, dolichol, heme and prenylated proteins. The third module or late pathway involves the ergosterol synthesis itself through consecutive reactions that mainly occur in the endoplasmic reticulum (ER) membrane. Firstly, two molecules of farnesyl-PP are used by the squalene synthase Erg9 enzyme to form squalene, which is the precursor of all steroids. Secondly, squalene is converted into lanosterol by the consecutive action of the squalene epoxidase Erg1 and the lanosterol synthase Erg7 enzymes. In the next steps, lanosterol is transformed to zymosterol through a complex process involving various demethylation, reduction and desaturation reactions catalyzed by the lanosterol 14-α-demethylase Erg11 (also known as Cyp51), the C-14 reductase Erg24 and the C-4 demethylation complex Erg25-Erg26-Erg27. Erg28 and Erg29 are likely to function in the C-4 demethylation complex reaction [8,13]. Zymosterol is the first intermediate of the biosynthesis pathway that can be incorporated into cellular membranes [14]. Then, Erg6 converts zymosterol into fecosterol, followed by the formation of episterol by Erg2, which is finally desaturated and reduced by Erg3, Erg5 and Erg4 to ergosterol. Ergosterol is synthesized in the ER, but is mostly transported to the plasma membrane (PM). Erg1 and Erg11 represent two rate-limiting steps in this part of the pathway. Ergosterol biosynthesis depends on both oxygen and iron in multiple enzymatic steps (Figure 1). Molecular oxygen is the electron acceptor in the enzymatic steps catalyzed by Erg1, Erg11, Erg25, Erg3 and Erg5; and heme, whose biosynthesis requires oxygen and iron, directly associates to Erg11 and Erg5, as well as to Erg25 and Erg3 as a cytochrome b5 cofactor (Figure 1). Moreover, Erg11 and Erg5 belong to the cytochrome P450 family of enzymes that function in association with regulators such as the P450 reductase Ncp1 or the heme-binding protein Dap1. Meanwhile, Erg25 and Erg3 are oxo-diiron enzymes of the fatty acid hydroxylase/sterol desaturase family (Figure 1). Thus, oxygen and iron depletion are associated with reduced activity of these enzymes and changes in sterol production.

Defects in ergosterol biosynthesis lead to impaired endocytosis, cell polarization, cell fusion and cell wall assembly (reviewed in [15]). The single deletion of the majority of ergosterol biosynthetic (*ERG*) genes within the late pathway is lethal under standard growth conditions without the addition of ergosterol. The only exceptions are the last five enzymes, encoded by the *ERG2* to *ERG6* genes, probably due to the similar physicochemical properties that the intermediates accumulated in these mutants exhibit with respect to ergosterol, and *ERG28*. Curiously, sterol profiling suggests that Erg2 to Erg6 enzymes display low substrate specificity and can accept a broad range of similar sterol structures. Thereby, their deletion leads to the accumulation of sterol mixtures instead of only their substrate in the pathway (reviewed in [16]). For instance, the *erg6* mutant accumulates its substrate and several sterols resulting from the catalytic activity of Erg2, Erg3 and Erg5 on zymosterol [17]. These *erg* mutants are defective in different cellular processes and show alterations in the resistance to certain stresses (reviewed in [16,18]). The deletion of one of the two isozymes encoded by *HMGR* (*HMG1* or *HMG2*) has only a little effect on cell growth, but the double mutant is inviable. Significantly, the overexpression of each *ERG* gene leads to a great variability in the tolerance to stressors and antifungal drugs, which reflects the specialization of each enzyme and the pleiotropic nature of ergosterol biosynthesis disruption [19].

The main targets for antifungal drugs are the enzymes that constitute the last ergosterol biosynthesis module (Figure 1). Allylamines are noncompetitive inhibitors of fungal Erg1 and are effective against dermatophyte infections [20]. Azoles, the most common drug used to treat fungal infections, target Erg11 by directly binding to the iron atom within the heme group of the enzyme [21]. When Erg11 is inhibited, an alternate pathway catalyzed by Erg6, Erg25-Erg26-Erg27 and Erg3 is activated leading to the formation of the fungistatic 14α methylergosta 8-24 (28) dienol [19,22]. Thus, mutations in *Candida albicans ERG6* and *ERG3* genes lead to the development of azole resistance [19,23]. Morpholines are used as agricultural fungicides via inhibition of two separate steps, Erg2 and, particularly, Erg24 [24]. Polyenes, including nystatin and amphotericin B, which are frequently used in the medical treatment of systemic infections, directly interact with cell surface ergosterol [25]. In addition, statins competitively inhibit human and fungal Hmg1, and are commonly used in humans to lower cholesterol levels. The only Erg enzymes that are not conserved in mammals are Erg6, Erg2, Erg5 and Erg4 (reviewed in [26]). Of them, both the deletion and the overexpression of *ERG6* provoke the most compromised phenotypes suggesting that it could be a suitable target for a new generation of antifungal agents [19,27].

### 2.2. Sterol Acquisition and Transport in S. cerevisiae

Ergosterol biosynthesis is a highly energy-consuming process that requires 24 molecules of ATP and 16 molecules of NADPH to obtain a single ergosterol compound (reviewed in [6]). Despite this, *S. cerevisiae* cells do not import significant amounts of extracellular sterols in the presence of oxygen, a phenomenon referred to as “aerobic sterol exclusion” [28]. It has been proposed that aerobic sterol exclusion could be a strategy to ensure that only the most adequate sterols are incorporated to membranes. The decreased ability to synthesize sterols under hypoxic or anaerobic conditions is compensated by the import of sterols from the medium. Different sterol uptake responses have been observed for fungi like *Schizosaccharomyces pombe*, *Candida glabrata* and *C. albicans* [29,30]. Due to their hydrophobicity, sterols are transported inside cells either in a vesicular manner or via lipid binding/transfer proteins. The last mechanism seems to be the prevailing one in *S. cerevisiae*, since the inhibition of vesicular transport through the secretory pathway does not significantly affect sterol transport to the PM (reviewed in [18]). In *S. cerevisiae*, sterol uptake initiates with the interaction between the external sterol and the cell wall, followed by its incorporation into the PM in an ATP-dependent manner by the PM ATP-binding cassette (ABC) transporters Aus1 and Pdr11 [31]. Then, sterol sphingolipid-enriched microdomains, called rafts, are formed, and sterols are progressively transported from the cell surface to the ER through a non-vesicular process also mediated by Aus1 and Pdr11 [32,33]. In the ER, sterols are partly esterified to be stored in lipid particles (LPs), and the non-esterified sterols are returned to the PM [32]. Sterol esterification is essential for efficient sterol uptake [32]. After shifting to aerobic conditions, newly synthesized ergosterol is likely to displace the exogenous sterols at the PM [34].

Despite the ER being the site of sterol biosynthesis, it contains very little ergosterol, which is mostly transported to other cellular membranes, especially the PM. Most intracellular sterol trafficking seems to take place through non-vesicular transport. Two families of evolutionary conserved sterol-binding proteins mediate intracellular sterol distribution: oxysterol-binding protein homologs (Osh1 to Osh7) and lipid transfer proteins anchored at membrane contact sites (Lam1 to Lam6) [35,36,37,38]. Another protein that is involved in sterol transport and exogenous sterol uptake is Arv1, which seems to catalyze the insertion of tail-anchored proteins with a single carboxy-terminal transmembrane domain into membranes [39,40,41].

### 2.3. Sterol Detoxification

Yeast cells can overproduce ergosterol or sterol intermediates, which can be incorporated into cellular membranes to some extent, to modulate their physicochemical properties. However, the accumulation of excess free sterol intermediates may become toxic for cells since it alters mitochondrial respiration, resulting in the generation of toxic methyl sterol intermediates, which raise mitochondrial oxidants and decrease the ability to synthesize iron–sulfur (Fe-S) clusters [13,42,43]. To prevent these harmful effects, yeast cells utilize multiple detoxification mechanisms. Sterols cannot be degraded, but are either stored in the form of steryl esters (SE) in LPs or secreted into the medium as sterol acetates (reviewed in [6,18]). Under aerobic conditions, SE formation in the ER is catalyzed mainly by Are2, which has a significant preference for ergosterol as a substrate, whereas Are1 performs this function in hypoxia [44]. Conversely, Yeh1 and Tgl1 catalyze SE hydrolysis in LPs, and Yeh2 in the PM [45,46,47]. Esterified sterol reserves can be interconverted into free sterols and mobilized when ergosterol levels decrease. Interestingly, the double mutant *are1∆are2∆* does not display any altered growth under normal conditions, despite overall sterol biosynthesis drops and the level of free sterols increases [44,48]. These findings suggest that sterol biosynthesis and SE formation are interconnected through a regulatory mechanism. In fact, it has been reported that the expression of *ERG3* is down-regulated and Erg1 protein is destabilized in the *are1∆are2∆* double mutant [48,49,50]. In another detoxification mechanism, sterols are acetylated by Atf2 in the ER and then transported to the PM, where they are secreted via Pry1 and Pry2 (pathogen-related yeast proteins) [51,52]. Under normal conditions, newly synthesized ergosterol is acetylated by Atf2 and then rapidly deacetylated by Say1 in the ER [51]. Besides detoxification, sterol acetylation also contributes to the elimination of damaged lipids.

## 3. Regulation of Ergosterol Biosynthesis

To alter the ergosterol composition of lipid bilayers and, consequently, to be able to properly adapt to particular environmental stresses, yeast cells have evolved different regulatory mechanisms that tightly control sterol levels. The maintenance of suitable ergosterol concentrations is achieved by feedback mechanisms at the transcriptional and post-translational levels.

### 3.1. Subcellular Localization of Ergosterol Biosynthesis Enzymes

Multiple enzymes that participate in the late stage of ergosterol biosynthesis are transmembrane-containing proteins that associate into a functional complex, denoted the ergosome, to facilitate catalysis [53]. Erg11, Erg25, Erg27 and Erg28 represent the core center of the complex that interacts with other enzymes of the pathway [53]. Although sterol synthesis takes place in the ER, various Erg enzymes (Erg1, Erg7, Erg27 and Erg6) can also localize to LPs [54,55], which are not only universal storage organelles for neutral lipids. The dual localization of sterol enzymes in both subcellular organelles is also observed in cholesterol biosynthesis [56]. The association and subcellular localization of Erg enzymes seems to determine their functionality, although its regulation is poorly characterized. Erg1 displays a dual localization at the ER and LPs, being enzymatically inactive in the latter [57]. When ergosterol synthesis decreases due to iron deficiency, Erg1 exclusively localizes to the ER, where it is active, probably to enhance the iron-limited production of ergosterol [9]. Erg7 also displays a dual ER and LP localization, whereas Erg11 and Erg24 localize to the ER membrane. Erg28 functions as a scaffold of the C-4 demethylation enzyme complex Erg25–Erg26–Erg27 tethering it to the ER, and acting as a bridge to the Erg6 enzyme required for the next biosynthetic step [58,59]. As indicated above, Erg27 can also localize to LPs, where it physically binds to Erg7, promoting its association with LPs and preventing its degradation [60]. Upon a block in mitochondrial respiration, Erg27 migrates rapidly from LPs to the ER, although this does not imply the relocation of Erg7 [4]. Interestingly, a fusion between Erg7 and the ER-resident protein Erg28 restores ergosterol biosynthesis to *erg27* mutants, suggesting that the retention of Erg7 in the ER increases its activity [61]. Interestingly, Erg6 is mostly located in LPs [57], but can also be found in the ER, the cytoplasm and mitochondria. Finally, Erg2, Erg3, Erg5 and Erg4 are located primarily in the ER, from where ergosterol is distributed to its final membranous destinations [44,55].

### 3.2. Post-Translational Feedback Regulation

A key checkpoint for ergosterol biosynthesis involves the synthesis of HMG-CoA, catalyzed by HMGR (reviewed in [62]). Given that HMGR acts at the initial steps of the pathway, its regulation alters the synthesis of all sterols but also of other essential mevalonate-derived metabolites, such as ubiquinone, dolichol or heme. In *S. cerevisiae*, excess of oxysterols promotes the degradation of the Hmg2 enzyme by the ER-related degradation (ERAD) pathway. This process does not require the typical ERAD factors; instead, Hmg2 ubiquitination is mediated by the membrane-spanning E3 ligase Hrd1 and the E2 ubiquitin conjugating enzyme Ubc7, and it is subsequently released from the ER to the cytoplasm, where it is degraded by the proteasome [63,64]. The yeast squalene monooxygenase Erg1 is also degraded through the ERAD pathway via the ubiquitin ligase Doa10 when lanosterol concentrations increase, in order to prevent the accumulation of toxic sterol intermediates (Figure 1) [65]. Both ERAD-dependent mechanisms seem to be conserved in mammalian cells.

### 3.3. Transcriptional Regulation

#### 3.3.1. Transcriptional Regulation by Sterols

**Figure 2:**
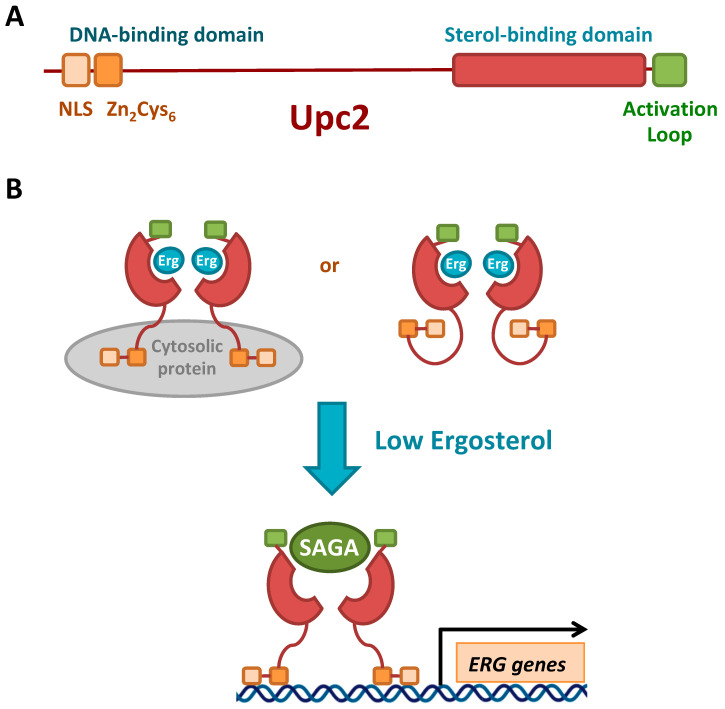
Structure and regulation of the *S. cerevisiae* Upc2 transcriptional factor. (**A**) Schematic representation of the primary structure of yeast Upc2 protein. NLS, nuclear localization signal. (**B**) Proposed model for the transcriptional regulation of Upc2. Under high-sterol conditions, Upc2 associates to ergosterol (Erg) and localizes to the cytosol. Although the mechanism is currently unknown, it has been proposed that Upc2 stays in the cytosol either because it interacts with a cytosolic protein or because its NLS is not available for nuclear import. Upon sterol depletion, ergosterol-free Upc2 undergoes a conformational change that allows its import to the nucleus, where it binds to *ERG* gene promoters to activate their transcription through the recruitment of a co-activator, probably the SAGA complex.

The expression of many enzymes within the ergosterol biosynthesis pathway is regulated at the transcriptional level by sterol abundance through the action of transcriptional factors that bind to 7-base pair DNA motifs, known as sterol regulatory elements (SREs), located in the promoter of their corresponding genes (reviewed in [26]). In mammals, the membrane-bound basic helix-loop-helix transcription factor sterol regulatory element binding protein (SREBP) is cleaved from the ER membrane by the SREBP cleavage activating protein (SCAP) and activated in response to sterol depletion. Most fungi, including the fission yeast *S. pombe* and the opportunistic pathogen *Cryptococcus neoformans* contain homologs of both SREBP and SCAP, known as Sre1 and Scp1, respectively [29,66]. SCAP and Scp1 constitute the sterol sensors in mammals and *S. pombe*, respectively [29,67]. Remarkably, the SREBPs from *S. cerevisiae* and the fungal commensal-pathogen *C. albicans* do not regulate sterol synthesis; instead they control filamentous growth. During *Saccharomycotina* evolution, a member of the fungus specific Zn_2_-Cys_6_ binuclear cluster family of transcription factors called Upc2 displaced SREBP proteins as the major sterol regulator [68,69,70]. Due to the whole genome duplication, *S. cerevisiae* also expresses a Upc2 paralog, known as Ecm22. Both transcription factors are able to bind, through their amino-terminal Zn_2_-Cys_6_ DNA-binding domain, to TATACGA SREs, which are identical to the anaerobic response elements AR1_c_ (Figure 2A) [68]. The recent elucidation of the structure of the carboxy-terminal domain (CTD) of yeast Upc2, which displays a novel α-helical fold, has demonstrated that it serves as an ergosterol-binding and sensing domain (Figure 2A) [71]. Under normal conditions, the binding of ergosterol to a deep hydrophobic pocket within Upc2 CTD retains the transcription factor in the cytosol in its repressed form, probably because sterol binding masks its bipartite amino-terminal nuclear localization signal (NLS) or because of its interaction with a hypothetical cytosolic protein (Figure 2B) [71,72]. Upon ergosterol depletion, the dissociation of the sterol ligand causes a conformational change that exposes Upc2 NLS, causing its translocation to the nucleus, where it activates the transcription of SRE/AR1_c_-containing genes including most *ERG* genes, sterol uptake genes (*AUS1*, *PDR11*), the *DAN*/*TIR* genes, which encode nine cell wall mannoproteins, and members of the seripauperin (*PAU*) gene family (Figure 2B) [68,71,72,73,74,75]. The conformational flexibility of the carboxy-terminal 30 amino acids of Upc2 constitute an activation loop that, in addition to sterol binding and the regulation of subcellular localization, contributes to transcriptional activation [71]. The dissociation of ergosterol from the Upc2 ligand-binding domain could also promote conformational changes that allow the activation loop to recruit downstream transcriptional Upc2 co-activators (Figure 2B). Recent data using an *erg3* mutant strain indicate that the SAGA co-activator complex is recruited to the promoter of *ERG* genes to promote their transcriptional activation [76]. Therefore, it has been proposed that the SAGA complex could be the co-activator that Upc2 recruits to enhance the expression of *ERG* genes when sterols abundance decreases, although no direct evidence exists (Figure 2B). Moreover, size-exclusion chromatography analyses strongly suggest that the biologically relevant form of Upc2 is a constitutive homodimer, which is assembled through its carboxy-terminal sterol-binding domain (Figure 2B) [71]. Dimerization is essential for Upc2 regulatory function, as its disruption traps the transcription factor in the cytosol [71]. In fact, this dimeric nature seems to be conserved because *C. albicans* Upc2 and *S. cerevisiae* Ecm22 ligand-binding domains also form dimers in solution [71]. Multiple gain-of-function mutations in *C. albicans* Upc2 CTD result in the constitutive activation of the ergosterol synthesis pathway and sterol uptake, which diminishes the strain susceptibility to azole drugs. Moreover, *UPC2* deletion increases *C. albicans* sensitivity to azole treatments, even in highly azole resistant clinical isolates containing multiple resistance mechanisms [70,77]. Therefore, Upc2 represents an important target to improve antifungal azole therapies.

**Figure 3:**
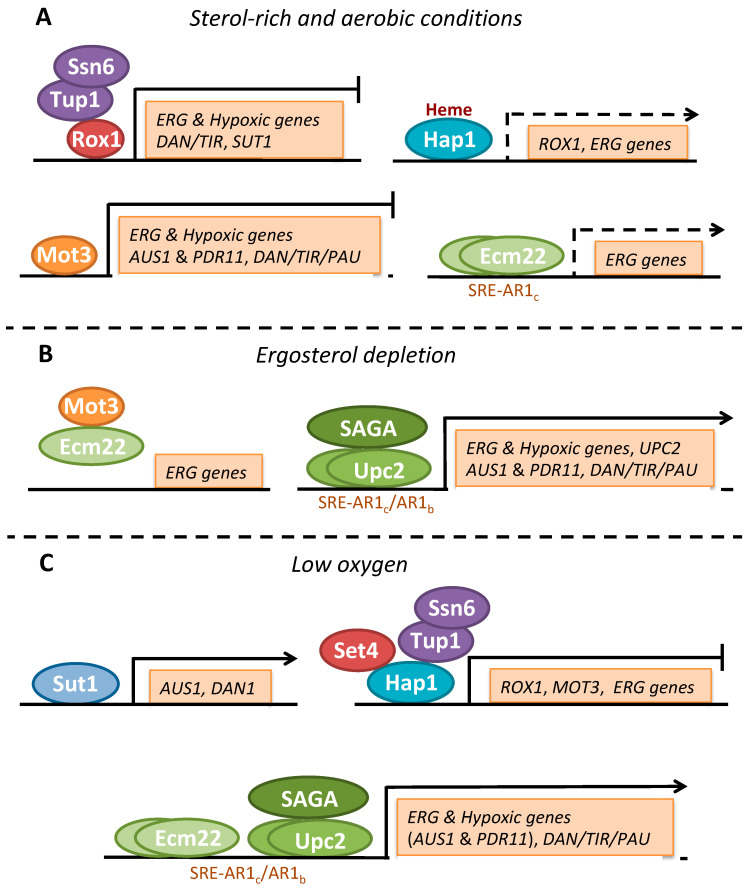
Transcriptional regulation of *S. cerevisiae* genes involved in ergosterol biosynthesis and uptake in response to low sterols or oxygen. (**A**) Under normal growth conditions, basal *ERG* gene expression is mostly maintained by Ecm22 and Hap1. Moreover, Mot3 and Rox1 (in a Tup1-Ssn6-dependent manner) inhibit the expression of hypoxic and ergosterol uptake genes. (**B**) Upon sterol depletion, Upc2 activates the transcription of *ERG* genes probably by recruiting the SAGA complex. (**C**) A drop in oxygen availability decreases heme levels, which lead to the Tup1-Ssn6 and Set4 recruitment by Hap1 to repress the expression of some *ERG* genes, *ROX1* and *MOT3*. The decrease in Rox1 and Mot3 levels increases the expression of *ERG*, hypoxic and sterol uptake genes in a Upc2-Ecm22-dependent manner. Finally, Sut1 activates the transcription of *AUS1* and *DAN1*, but not *PDR11*.

Under normal laboratory growth conditions, Ecm22 and, to a lesser extent, Upc2 bind to *ERG* gene promoters to maintain a basal expression level (Figure 3A) [68]. Upon low sterol levels, the abundance of Ecm22 protein, and consequently its binding to *ERG* promoters, decreases in a process that involves its direct physical interaction with the repressor Mot3 (Figure 3B) [74,75]. At the same time, the levels of Upc2, and their association to *ERG* promoters, strongly increase, which finally results in the transcriptional induction of *ERG* genes [68,74]. Interestingly, an auto-induction of *UPC2* transcription, exclusively mediated by the direct binding of Upc2 to two TAAACGA anaerobic response (AR1_b_) elements within its own promoter, is required not only for the increase in Upc2 protein abundance, but also for the global activation of *ERG* genes and the resistance to antifungal drug treatments (Figure 3B) [78]. In addition to *UPC2*, *ERG* genes including *ERG3* and *ERG25* contain AR1_b_ motifs essential for their correct antifungal-induced expression through Upc2 [78]. The specific in vitro and in vivo binding of Upc2 proteins from *S. cerevisiae*, but also from the pathogenic fungi *C. albicans* and *C. glabrata*, to SRE/AR1_c_ and AR1_b_ elements with similar affinities reinforces this regulatory mechanism [78]. However, not all SRE/AR1_c_ or AR1_b_ consensus sequences seem to be functional in vivo [78]. In mammals, SREBPs have also been shown to bind promoters containing sequences other that the SRE consensus.

Other factors beyond Upc2 and Ecm2 contribute to the activation of *ERG* genes. The heme-dependent transcription factor Hap1 is also necessary for the expression of genes involved in ergosterol biosynthesis (Figure 3A) [75,79,80,81,82]. Genetic data suggest that Hap1 is required for the basal expression of some *ERG* genes and for their Ecm22-dependent activation by sterol depletion [75]. In fact, the deletion of both *UPC2* and *ECM22* is synthetically lethal in the S288C yeast genetic background due to a Ty1 insertion mutation into *HAP1* coding sequence that partially compromises its function, while it is viable in the W303 background with a wild-type *HAP1* gene [75,83]. Consistent with these observations, yeast strains with a *HAP1*-inserted transposon have less cellular ergosterol content [80]. Finally, Mga2, the transcription factor that controls the expression of the ∆9 fatty acid desaturase Ole1, which is responsible for the synthesis of unsaturated fatty acids, is required for the full basal expression of *ERG1* [84]. As a matter of fact, *mga2∆* cells accumulate higher relative levels of squalene than wild-type cells, suggesting a coordinated regulation between fatty acid and sterol biosynthesis [84]. In *S. pombe*, SREBP Sre1 activation requires unsaturated fatty acid synthesis, and sterol biosynthesis is necessary for Mga2 transcriptional activity [85]. These observations highlight the connections between ergosterol and fatty acids metabolisms to modulate membrane properties.

#### 3.3.2. Transcriptional Regulation by Oxygen

As mentioned above, ergosterol biosynthesis depends on oxygen and heme as cofactors for critical enzymes of the pathway (Figure 1). During aerobic growth, heme is properly synthesized and available for binding to Hap1 protein. Under these conditions, heme-bound Hap1 not only activates the expression of *ERG* genes, but also of the gene coding for the DNA-binding protein Rox1, which represses, in a Tup1-Ssn6-dependent manner, the transcription of a group of genes referred to as “hypoxic genes”, which encode intracellular proteins that allow an efficient utilization of oxygen (e.g., *ANB1* and *HEM13*), as well as the *DAN*/*TIR* genes and *UPC2* [86,87,88,89,90] (Figure 3A). In addition, to limit Ecm22 function [75], the transcriptional factor Mot3 also represses the expression of hypoxic, *DAN*/*TIR/PAU* and *ERG* genes including *ERG2*, *ERG6* and *ERG9* (Figure 3A) [86,89,90,91,92,93]. Rox1 and Mot3 regulatory factors act synergistically to achieve stringent repression of target genes [89,90]. As a general result, under aerobic conditions, *ERG* genes display basal levels necessary for ergosterol biosynthesis, whereas the hypoxic-responsive genes are fully repressed. Conversely, a decrease in oxygen bioavailability causes a drop in heme and ergosterol levels that triggers the activation of signaling pathways that induce the expression of both hypoxic and *ERG* genes [94]. The up-regulation of oxygen-dependent enzymes within the ergosterol biosynthesis pathway upon oxygen limitation could be considered as a cellular strategy to compensate for their decrease in activity. Although both pathways are interdependent, heme depletion mainly signals the Hap1 pathway, while sterol depletion directly activates the expression of *ERG* and *DAN/TIR/PAU* genes via Upc2-Ecm22 (Figure 3C) [75,86,95]. In addition to Upc2, the mitogen-activated protein kinase (MAPK) Hog1 and specific components of the SAGA co-activator complex also contribute to the induction of *PAU* genes in response to oxygen limitation [96]. Oxygen depletion also enhances the expression of the oxygen-dependent fatty acid desaturase Ole1 through the Mga2 transcriptional factor [97]. Importantly, Hap1 seems to shift from an activator to a transcriptional repressor when oxygen availability decreases [98]. Thus, under low oxygen conditions, heme-free Hap1 recruits the Tup1-Ssn6 general co-repressor complex and the SET domain-containing epigenetic factor Set4 to repress many *ERG* genes (including *ERG2*, *ERG3*, *ERG5* and *ERG11*), *ROX1* and *MOT3* [89,98,99], which in turn facilitates Upc2 and Ecm22 activation and the derepression of the hypoxic genes (Figure 3C) [75,87]. However, conflicting results have been observed for the effect of Hap1 on the expression of *ERG* genes during anaerobic conditions [75,96,99,100]. These discrepancies could be due to the use of yeast strains expressing different Hap1 factors, or being cultivated in media with different oxygen, ergosterol or fatty acid availability [29,96,100].

Many aspects of their regulation are still unknown due to the crosstalk between different environmental signals and the opposite regulatory effects triggered by Hap1, Rox1, Mot3, Upc2 and Ecm22 regulatory factors on a large number of *ERG* genes. Several genome-wide expression studies based on microarray and RNA-Seq analyses under low oxygen conditions have reflected that the pattern of expression differs among *ERG* genes, although some general trends can be extracted [94,96,98,100,101,102,103]. *ERG* genes involved in the first two modules of the ergosterol biosynthesis pathway seem to be down-regulated (e.g., *ERG8*, *ERG13* and *ERG19*) or constitutively expressed (e.g., *ERG10* and *IDI1*) when oxygen availability decreases, whereas most genes involved in the latter module are induced (e.g., *NCP1*, *DAP1*, *ERG25, ERG26*, *ERG28*) and only few of them are down-regulated (e.g., *ERG5*). As expected, the number of *ERG* genes with altered expression increases with the severity of oxygen restriction, and genes encoding for oxygen-dependent enzymes are up-regulated (e.g., *ERG1*, *ERG11* and *ERG3*) upon acute oxygen starvation conditions [94,102,103]. Different hypotheses have been proposed to explain these observations. The increase in the expression of oxygen-using enzymes may be taking place to maintain the flux of ergosterol formation. However, the pattern of *ERG5* expression contradicts this hypothesis, since it is down-regulated. Another possibility is that only the expression of particular *ERG* genes is enhanced to prevent the accumulation of toxic sterol intermediates. The up-regulation of the latter module of the ergosterol biosynthesis pathway under severe low oxygen conditions could favor the rapid production of ergosterol upon reoxygenation [94,102,103]. In accordance with this hypothesis, some of the genes that are most strongly induced upon reoxygenation are involved in the two first modules of ergosterol synthesis [103]. This could be important during anaerobic-to-aerobic transitions, since de novo sterol synthesis is required for the induction of respiratory genes [104]. Further studies are necessary to fully elucidate the regulation of *ERG* genes upon oxygen limitation.

As already mentioned, yeast cells activate sterol import under anaerobic conditions. By using a hyperactive *upc2-1* allele, the cell wall mannoprotein Dan1 was identified as a facilitator of sterol influx, in addition to Aus1 and Pdr11 [73]. In fact, the overexpression of *DAN1* and *AUS1* is sufficient to promote the uptake of sterol in aerobiosis [105]. When the levels of oxygen are appropriate (normoxia), Mot3 represses *AUS1* and *PDR11* expression [106], whereas Upc2 allows their up-regulation upon oxygen depletion [73]. Additionally, the transcription factor Sut1, which is up-regulated under anaerobiosis due to loss of Rox1 repression [94], stimulates the expression of *AUS1* and *DAN1*, but not *PDR11* (Figure 3C) [105]. As well as the *upc2-1* mutation, the constitutively active mutant of Ecm22 imports sterols under normoxia, but the underlying mechanisms are poorly characterized [107]. Under oxygen limitation, *DAN/TIR/PAU* genes replace the major aerobic cell wall mannoproteins encoded by *CWP1* and *CWP2*. As mentioned above, the hypoxic expression of *DAN/TIR/PAU* genes depends on both sterol and heme levels due to Rox1-Mot3 derepression and Upc2-Ecm22-mediated activation (Figure 3C) [75,89,91,93,95]. However, the relative importance of each factor differs and depends on each particular *DAN/TIR* gene. Thus, *DAN2* and *DAN4* are not regulated by Rox1 and seem to be primarily induced by low sterols [86], while *DAN1* and *TIR1* respond to the depletion of both metabolites [75,95].

#### 3.3.3. Transcriptional Regulation by Osmotic Stress

The response and adaptation to hyperosmotic stress is mostly governed by the high osmolarity glycerol (HOG) pathway and its terminal signaling MAPK Hog1 (reviewed in [108]). Upon osmostresses such as high extracellular salt concentrations, Hog1 induces the transcription of *MOT3* [109], leading to a transient increase in Mot3 protein abundance that facilitates its association to particular *ERG* promoters [7]. As a result, there is a rapid and transient down-regulation of genes related to sterol biosynthesis (*ECM22*, *ERG2* and *ERG11*), sterol uptake (*SUT1* and *AUS1*) and cell wall components (several *DAN*/*TIR* and *PAU* genes) that causes a decrease in cellular ergosterol content that is physiologically relevant for osmostress adaptation [7,110]. Consistent with this, the expression of the hyperactive *upc2-1* allele increases ergosterol content and renders cells highly salt sensitive, whereas inhibition of ergosterol synthesis with azole drugs is beneficial for salt-stress tolerance [7]. Both *mot3∆* and *rox1∆* mutants accumulate Na^+^ upon salt stress [7], suggesting that ergosterol down-regulation may be important for the appropriate regulation of plasma membrane ion transporters such as the Na^+^-ATPase Ena1 or the H^+^-ATPase Pma1, although further studies are required to decipher their involvement.

### 3.4. Regulation by Iron Bioavailability

As already indicated, ergosterol biosynthesis depends on iron in four steps, which are catalyzed by enzymes that contain a cofactor in the form of heme (Erg5, Erg11 and its regulator Dap1) or oxo-diiron (Erg25 and Erg3) centers (Figure 1). Consequently, iron deficiency reduces the metabolic flux through the sterol pathway, leading to a decrease in ergosterol and zymosterol levels, and the accumulation of squalene and lanosterol [9], which are the substrates of Erg1 and Erg11, respectively. Erg11 function is likely to decrease due to the drop in heme levels that occurs when iron is scarce, and the subsequent lanosterol accumulation may inhibit Erg1 [9,65]. Further studies have indicated that the heme-binding domain of the cytochrome *b_5_* related protein Dap1 is required for the activity of the cytochrome P450 enzyme Erg11 as well as for growth in iron-deficient conditions [111]. Although it has been proposed that Dap1 increases Erg11 protein abundance in a heme-dependent manner, the regulation of Erg11 by Dap1 has not been fully deciphered [112,113]. In any case, under iron deprivation, the loss of Dap1 is rescued by *ERG11* overexpression but not by increasing heme biosynthesis [111]. In addition to Dap1, mutants in other components of ergosterol biosynthesis, such as in the essential genes *ERG25* and *ERG29*, also lead to growth defects in low iron and respiratory conditions, respectively, due to impaired ergosterol production [114,115]. A recent study has revealed that, similarly to Erg25, Erg29 participates in the methyl sterol oxidase step of ergosterol biosynthesis [13]. Defects in this step lead to the accumulation of toxic intermediates of the methyl sterol oxidase reaction that increase mitochondrial oxidation and affect the stability of the yeast frataxin homolog Ffh1, which is implicated in mitochondrial iron metabolism [13]. As a consequence, *erg29* mutants exhibit defects in iron-sulfur cluster assembly and mitochondrial iron accumulation [13,115]. These results emphasize the multiple connections between iron metabolism and ergosterol biosynthesis.

Genome-wide expression studies have shown that the expression of *ERG* genes is altered in response to iron deficiency [116,117]. Under iron limitation, yeast cells lacking the iron-regulated RNA-binding protein Cth2 display high mRNA levels of multiple *ERG* genes including those of the first steps of the late stage of synthesis (*ERG1*, *ERG7*, *ERG11* and *DAP1*) [116,118]. Upon iron starvation, Cth2 protein binds to multiple mRNAs containing AU-rich elements (AREs) within their 3′ untranslated region (3′UTR), and promotes their degradation and translation inhibition [116,119,120]. The presence of multiple AREs on the 3′UTR of this subset of *ERG* genes suggests that Cth2 may trigger their down-regulation when iron is scarce. Consistent with this, the protein levels of both Erg1 and Erg11 decrease in response to iron depletion [9]. However, the abundance of other Erg proteins, such as Erg6 and Erg25, is kept roughly constant or even increases, which is the case of Erg3 [9]. In this sense, a recent global kinetic study of gene expression during the progress of iron deficiency has shown that the pattern of expression differs among *ERG* genes and depends on the severity of the depletion [121]. Therefore, we hypothesize that additional regulatory factors, including Hap1, Upc2, Ecm22 and others may directly or indirectly respond to iron limitation to control *ERG* gene expression and ergosterol synthesis.

## 4. Conclusions

The yeast *S. cerevisiae* is used as a reliable model to study multiple aspects of lipid biology due to its well characterized genome, the relative simplicity of its lipid metabolism and homeostasis, and the conservation of genes, routes and networks in other eukaryotes (reviewed in [122]). In recent years, ergosterol biosynthesis has been greatly studied in *S. cerevisiae*, leading to the identification of many conserved but also fungal-specific steps.

The balanced regulation of all the enzymes in the ergosterol biosynthesis pathway is an essential determinant of the efficiency of sterol synthesis and, therefore, of optimal growth and adaptation to environmental cues. Moreover, one of the main approaches to overproduce sterols of high-value for food and pharmaceutical industries includes yeast genetic modification. However, changes in ergosterol biosynthesis lead to pleiotropic defects that limit cellular proliferation and adaptation to stresses. Because of these reasons, the study of ergosterol biosynthesis regulation may provide new ideas for enhancing sterol production and the adaptation of yeast cell factories to the environment. Furthermore, chemicals could be developed to increase the production of specific sterols during yeast fermentation. For instance, the treatment with terbinafine, which targets the squalene epoxidase Erg1, results in the accumulation of squalene, which can be used for the synthesis of terpenes. In this sense, the genetic alteration of sterol metabolism could reduce the concentration of these expensive chemicals, drastically lowering production costs.

The ergosterol biosynthetic pathway constitutes one of the main targets for antifungal agents in health and agriculture. Nevertheless, the currently available drugs have been related to emerging resistance by fungal pathogens, significant side effects and toxicity (reviewed in [123]). The detailed study of the molecular mechanisms that contribute to the regulation, synthesis and transport of sterols in *S. cerevisiae* and fungal pathogens is crucial for the design of new antifungal strategies. Ergosterol regulation by particular metabolites and environmental cues is still far from being understood in all its complexity. Future lines of investigation should include the identification of factors that regulate the subcellular localization and function of Erg proteins and the structural and functional characterization of the fungal-specific zinc-finger regulatory proteins that control the expression of *ERG* genes.
